# ICF-based prediction of return to work after trauma rehabilitation: Results of the icfPROreha study in patients with severe musculoskeletal injuries

**DOI:** 10.3389/fresc.2022.960473

**Published:** 2022-09-01

**Authors:** Sandra Kus, Cornelia Oberhauser, Stefan Simmel, Michaela Coenen

**Affiliations:** ^1^ Institute for Medical Information Processing, Biometry, and Epidemiology–IBE, Chair of Public Health and Health Services Research, LMU Munich, Munich, Germany; ^2^ Pettenkofer School of Public Health, Munich, Germany; ^3^ ICF Research Branch, Nottwil, Switzerland; ^4^ Department for Rehabilitation, BG Hospital Murnau, Murnau, Germany

**Keywords:** biopsychosocial, musculoskeletal injury, trauma, return to work, rehabilitation, International Classification of Functioning, Disability and Health (ICF), assessment

## Abstract

**Background:**

Physical aspects such as the type and severity of an injury are not the only factors contributing to whether or not a person can return to work (RTW) after a serious injury. A more comprehensive, biopsychosocial approach is needed to understand the complexity of RTW fully. The study aims to identify predictors of RTW 78 weeks after discharge from initial inpatient trauma rehabilitation in patients with severe musculoskeletal injuries using a biopsychosocial perspective.

**Methods:**

This is a prospective multicenter longitudinal study with a follow-up of up to 78 weeks after discharge from trauma rehabilitation. Data on potential predictors were collected at admission to rehabilitation using a comprehensive assessment tool. The status of RTW (yes vs. no) was assessed 78 weeks after discharge from rehabilitation. The data were randomly divided into a training and a validation data set in a ratio of 9:1. On the training data, we performed bivariate and multiple logistic regression analyses on the association of RTW and potential predictors. The final logit model was selected *via* stepwise variable selection based on the Akaike information criterion. The final model was validated for the training and the validation data.

**Results:**

Data from 761 patients (*n* = 561 male, 73.7%; mean age: 47.5 years, SD 12.3), primarily suffering from severe injuries to large joints and complex fractures of the large tubular bones, could be considered for analyses. At 78 weeks after discharge, 618 patients (81.2%) had returned to work. Eleven predictors remained in the final logit model: general health, current state of health, sensation of pain, limitations and restrictions in activities and participation (disability), professional sector, ongoing legal disputes, financial concerns (assets), personality traits, life satisfaction preaccident, attitude to life, and demand for pension claim. A predicted probability for RTW based on the multiple logistic regression model of 76.3% was revealed as the optimal cut-off score based on the ROC curve.

**Conclusion:**

A holistic biopsychosocial approach is needed to address RTW and strengthen person-centered treatment and rehabilitation. Patients at risk for no RTW in the long term can already be identified at the onset of rehabilitation.

## Introduction

The World Health Organization (WHO) reports intentional and unintentional injuries as the leading cause of death and disability, accounting for nearly 500,000 deaths in the WHO European Region in 2016 (1). Further, WHO points out that the number of deaths does not represent the full extent of the problem and that nonfatal injuries leading to disabilities that impact peoples' lives pose a massive burden on health care systems. It is estimated that for every death, 166 people are injured each year, resulting in over 38 million injuries in the EU each year (1).

In Germany, almost 1.7 million people with injuries are treated in hospitals per year (2). The 2020 annual report of the TraumaRegister DGU® of the German Society for Trauma Surgery (DGU) shows that about 30,000 people suffer a serious injury each year, according to the serious injury definition of the TraumaRegister DGU® (3). According to current data, the mortality rate of trauma patients in Germany is 10.3% over 10 years (3). Thus, the question is no longer only whether a person survives a serious accident and the associated trauma, but how they survive, and more specifically, whether a return to work (RTW) is possible and which factors determine RTW.

In the scientific literature, there is a lack of consistent evidence regarding associated factors for predicting RTW after traumatic injuries. In addition to injury severity and physical impairments, both personal and sociodemographic aspects as well as social and mental factors appear to have an influence on RTW (4–15). Some studies take a holistic view and consider the injured person with their professional, social, and personal background (8, 11, 16). However, these studies differ in terms of their methodological and conceptual implementation, as well as in selecting the analyzed influencing factors (predictors) (8).

The International Classification of Functioning, Disability and Health (ICF) (17), with its biopsychosocial perspective, offers a framework that enables a holistic view. In addition to body functions (e.g., pain) and structures (e.g., structures of the spine), activities (e.g., self-care), and participation (e.g., participation in social life)—subsumed in the ICF under “functioning”—the biopsychosocial perspective also includes “contextual factors” with environmental factors (e.g., social support) and personal factors (e.g., age). So far, there is a lack of a scientific study systematically taking into account a comprehensive and holistic concept, as provided by the ICF, in the selection, recording, and analysis of factors influencing RTW of severely injured persons.

This paper presents the results of a longitudinal study—conducted as part of the icfPROreha project—that aims to identify predictors of RTW 78 weeks after discharge from initial inpatient trauma rehabilitation in patients with severe musculoskeletal injuries using a biopsychosocial perspective. The results of our study are based on a broad database with a comprehensive survey of potential predictors of RTW generated in 10 hospitals across Germany. They provide valuable insights that considering a biopsychosocial perspective is important for understanding RTW. In addition, our research sheds light on which functioning aspects and contextual factors are important for predicting RTW. These findings can help professionals working in rehabilitation to become aware of potential cases with a risk of delayed RTW at an early stage and to take the appropriate action.

## Materials and methods

### Study design

The research project “ICF-based prediction of outcomes in rehabilitation after trauma—icfPROreha” (www.icf-proreha.de) is a joint project of the Chair of Public Health and Health Services Research–IBE of LMU Munich, the BG Rehabilitation Department of BG Unfallklink Murnau, and further nine cooperating rehabilitation departments all over Germany. The project aims to identify determinants (aspects of functioning and contextual factors) predicting RTW, time off work, and quality of life in persons with severe musculoskeletal injuries after inpatient trauma rehabilitation. In addition, an outcome prognosis and guidelines including recommendations have been established in icfPROreha, showing how to address the identified predictors in rehabilitation management to considerably reduce time off work and ensure a successful return to work.

**Figure 1:**
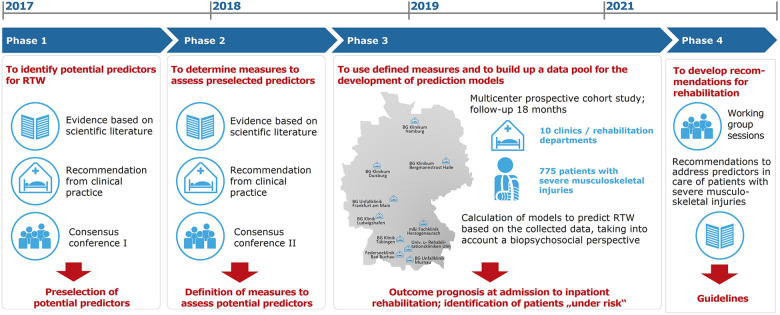
Project phases of icfPROreha.

The implementation of icfPROreha took place over 55 months (April 2017 to October 2021) and was divided into four phases (Figure 1).

In phase 3, we conducted a multicenter longitudinal study in collaboration with the participating rehabilitation departments in which functioning aspects, contextual factors, and injury-specific and sociodemographic data were systematically recorded using a comprehensive ICF-based assessment tool. The assessment tool provides a database for carrying out predictive analyses of the outcome RTW 78 weeks after discharge from inpatient trauma rehabilitation.

The study and its protocol were approved by the Ethical Committee of the Medical Faculty of LMU Munich (Project number: 18-329, 27 June 2018) and the Ethical Committees of the participating rehabilitation departments. It was registered in the German Register of Clinical Studies (DRKS-ID: DRKS00014857). All included patients gave written informed consent to participate in the study.

### Sample

The study included severely injured persons being admitted as inpatients to rehabilitation departments of one of the participating hospitals from August 2018 to December 2019 and met the following inclusion criteria: (1) age 18–65 years, (2) diagnosis of severe musculoskeletal injury according to the German injury classification, such as severe injuries to large joints (see Table 1) (18), (3) first inpatient trauma rehabilitation following accident or injury, (4) admission to inpatient rehabilitation within 16 weeks after accident or injury, (5) aim and conduct of the study were understood, and (6) a signed informed consent was obtained. Persons with injuries of the major nerve tracts, including spinal injuries with neurological symptoms and patients with craniocerebral injuries (from SHT grade II), were excluded.

**Table 1:** Sociodemographic and injury-specific data of the study population at admission to inpatient rehabilitation (t1) (*n* = 761).

Sociodemographic and injury-specific data	*n (*%)
Gender	
Male	561 (73.7)
Education	
No graduation	26 (3.4)
Graduation up to grade 9	274 (36.0)
Graduation up to grade 10	264 (34.7)
Graduation up to grade 12	197 (25.9)
Vocational training	
Completed vocational training	670 (88.0)
Cultural background	
Native language(s)—German	709 (93.2)
Social Status	
(a) Income (monthly household net income (€))	
Below 1700	140 (18.4)
1,700–2,300	160 (21.0)
2,300–3,200	152 (20.0)
3,200 and more	216 (28.4)
(b) Main earner	
Yes	315 (41.4)
No	287 (37.7)
No information	159 (20.9)
Employment status	
(a) Situation pre-accident	
Part-time employed	88 (11.6)
Full-time employed	659 (86.6)
Not working	14 (1.8)
(b) Employment type	
Self-employed	74 (9.7)
Dependent employed	687 (90.3)
Type of injury	
Extensive or deep injuries of the skin and soft tissue mantle; amputation injuries; muscle compression syndromes (compartment syndromes); thermal or chemical damage	43 (5.7)
Injuries to the great vessels	5 (0.7)
Severe chest or abdominal injuries with organ involvement including kidneys or urinary tract	60 (7.9)
Complex fractures of the large tubular bones, especially multiple or open fractures	235 (30.9)
Severe injuries to large joints	395 (51.9)
Severe injuries to the hand	44 (5.8)
Complex fractures of the facial skull and torso skeleton	199 (26.2)
Multiple injuries with severe manifestations	50 (6.6)
Localisation of injury	
Head (without facial skull)	20 (2.6)
Facial skull / face	28 (3.7)
Neck (spine)	11 (1.5)
Thorax	80 (10.5)
Abdomen	18 (2.4)
Back/spine (thoracic or lumbar spine)	102 (13.4)
Upper extremity (including shoulder)	250 (32.9)
Lower extremity (including hip and pelvic bones)	569 (74.8)
Type of accident—work or leisure accident	
Work accident	695 (91.3)

### Materials

In the prospective longitudinal study, a comprehensive assessment tool was used, which was developed by a group of experts in phases 1 and 2 of the project (Figure 1). A total of 47 potential predictors allotted to 82 variables were included in the analyses to identify the predictors of RTW 78 weeks after discharge from rehabilitation (Table 2). The following standardized questionnaires were used to record individual potential predictors: limitations and restrictions in activities and participation using the WHO Disability Assessment Schedule (WHODAS) 2.0 (19), emotional functions using the Patient Health Questionnaire (PHQ-4) (20), addictive behavior—alcohol (mis)use using the Alcohol Use Disorders Identification Test (AUDIT-C) (21), self-efficacy using the General Self-Efficacy Short Scale (ASKU) (22), resilience using the Resilience Scale (RS-13) (23), personality traits using the Big Five Inventory (BFI) (24), and current state of health using the EuroQol five-dimension (EQ-5D-5L) visual analogue scale (EQ-VAS) (25).

**Table 2:** Overview of variables (potential predictors).


Variables (potential predictors)
Health problem
General health
Current state of health
Pre-existing conditions (comorbidity)
Type of injury
Severity of injury
Timely diagnosis
Complications in healing process
Addiction behavior
Functioning
Energy and drive functions
Emotional functions
Sensation of pain
Functions of the cardiorespiratory system
Structure of upper extremity
Looking after one's health
Limitations and restrictions in activities and participation (disability)
Environmental Factors
Type of accident—work or leisure accident (nc)
Professional sector (e590)
Ongoing legal disputes (e550)
Treatment: time from accident to admission to inpatient rehabilitation (e580)
Treatment: time from end of acute treatment to onset of post-acute treatment (e580)
Treatment: Type of post-acute treatment (e580)
Information about injury and prognosis by healthcare professionals (e355)
Availability of case management / coordination (e580)
Financial concerns (assets) (e165)
Social insurance benefits (e570)
Support by family and friends (e310, e315, e325)
Support from professional environment (employer, colleagues) (e325, e330, e335)
Stressful life events (nc)
Personal Factors
Age at admission
Gender
Family situation
Education
Vocational training
Cultural background
Social status
Employment status
Subjective prognosis on RTW
Demand for pension claim
Inability to work before the accident
Personality traits
Self-efficacy
Attitude to life (work as an important purpose in life)
Appraisal of the consequences of the accident
Life satisfaction pre-accident
Resilience
Coping/dealing with the injury
Disease gain

### Data collection

Patients who met the inclusion criteria and who gave written informed consent to participate in the study were included in the study. They completed the assessment tool for recording the potential predictors up to 3 days after admission (t1) to initial inpatient rehabilitation in electronic form on a mobile device (tablet). Follow-up surveys on the status of RTW took place 12 (t3), 26 (t4) 52 (t5), and 78 weeks (t6) after discharge. Time points t3–t6 were conducted as telephone interviews; the end of data collection was 31 August 2021. In addition, the status of RTW was validated and—in case of missing values—complemented by routine data of the corresponding insurers. Only data on t1 (admission to rehabilitation) and t6 (78 weeks after discharge) were used here.

### Data analyses

In the first step, all cases with a known status of RTW 78 weeks after discharge from inpatient rehabilitation were identified (*n* = 761). In these analyses, we considered whether the patient—as a result of the accident—has RTW or not (yes vs. no). Further aspects, such as type of job (full-time or part-time) or type of work activity (previous activities or modified activities), were not taken into account. Individual missing values in the potential predictor variables were replaced by mode or median based on the data of the finally included patients (*n* = 775). Initial descriptive analyses were performed.

In the second step, we randomly divided the data into a training data set with 90% of patients to develop a regression model to predict RTW and a validation data set with 10% of patients to validate this regression model. Afterward, we investigated whether these two data sets have similar distributions in the predictors and the outcome of interest.

On the training data set, we conducted bivariate analyses on the association of RTW (dependent variable) and potential predictors. For the dependent variable, both bivariate regression models (logit models) and bivariate regression trees (classification trees) were calculated, i.e., models with a single predictor each. A number of metric variables were recoded based on the split values of the classification trees. Subsequently, we preselected potential predictors to be included in the multivariable analysis if they were associated with the dependent variable, i.e., showed a significance level of <0.1 for the bivariate logit models or <0.05 for the bivariate classification trees.

Next, a multiple logistic regression analysis was performed on the training data set—including all preselected potential predictors—in which the final logit model was selected via stepwise variable selection based on the Akaike information criterion (AIC) (26). Afterward, the final model was validated for the training data set and the validation data set, respectively. We calculated sensitivity (proportion of patients correctly identified as patients with no RTW), specificity (proportion of patients correctly identified as patients with RTW), positive predictive value (PPV: proportion of individuals who were predicted not to RTW and indeed did not RTW), and negative predictive value (NPV: proportion of individuals who were predicted to RTW and indeed did RTW). Two different cutoff scores on the predicted probability for RTW based on the multiple logistic regression model were compared: (1) the optimal cutoff score based on the receiver operating characteristic (ROC) curve, and (2) an alternative one to obtain a high sensitivity (at least 80% on the training data set) to identify at-risk individuals with increased priority.

## Results

### Study population

**Figure 2:**
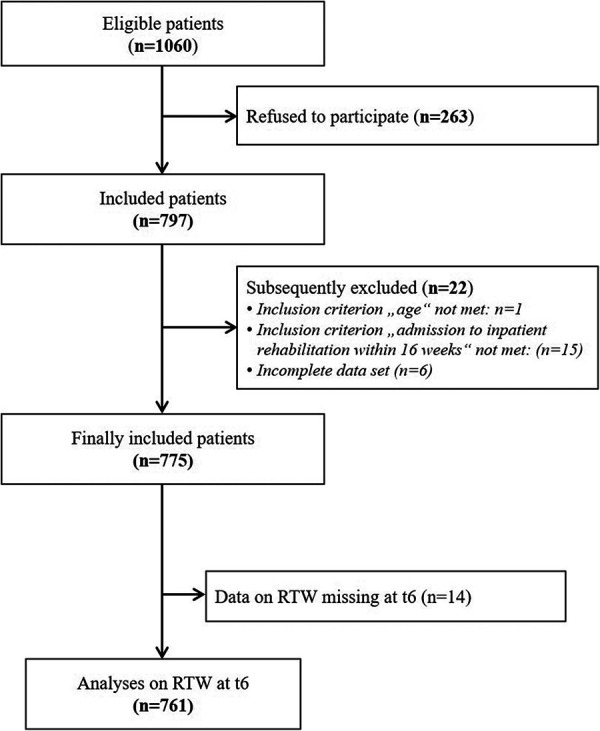
Flow chart of included and excluded patients and observations included in the analyses.

Of the 775 patients finally included in the study, data from 761 patients (*n* = 561 male, 73.7%; mean age: 47.5, SD 12.3) could be considered for this publication (Figure 2). Table 1 presents the results of the descriptive analyses on selected characteristics of the population.

According to the diagnoses, severe injuries to large joints (*n* = 395, 51.9%), followed by complex fractures of the large tubular bones (*n* = 235, 30.9%), were the most common injuries sustained. On average, patients were admitted to the first inpatient rehabilitation 37.4 days after discharge from the acute hospital, which lasted on average 6.2 weeks (median: 4.7 weeks).

#### Return to work

At 78 weeks after discharge from rehabilitation, 618 patients (81.2%) had returned to work, whereas 143 patients (18.8%) had not returned to work. Of the 618 patients with RTW at the observed point in time, about half had returned within 16 weeks after discharge from the first inpatient rehabilitation (median: 111.5 days; mean: 165.7 days; SD: 141.2). In terms of gender, the proportion of women having returned to work was 5.1% higher (*n* = 170; 85.0%) than the proportion of men (*n* = 448; 79.9%). However, bivariate analysis revealed no significant differences in RTW with regard to gender (*p* = 0.2482); thus, gender was not included in the multiple logistic regression analysis.

#### Prediction of return to work 78 weeks after discharge from rehabilitation

Of the 47 potential predictors of RTW, the following 11 predictors remained in the final logit model: general health, current state of health, sensation of pain, limitations and restrictions in activities and participation (disability), professional sector, ongoing legal disputes, financial concerns (assets), personality traits, life satisfaction preaccident, attitude to life, and demand for pension claim.

**Figure 3:**
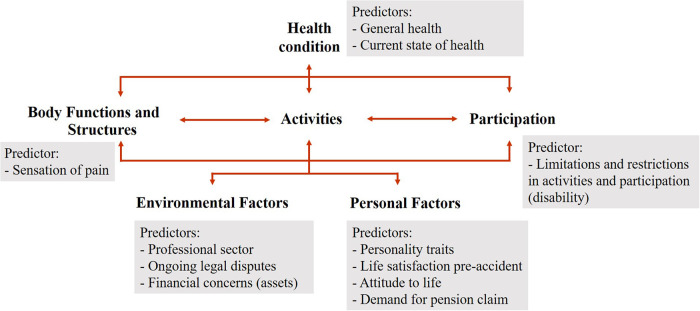
Assignment of predictors to the biopsychosocial perspective of the ICF.

The identified predictors comprise aspects related to the health problem and functioning aspects and contextual factors, i.e., they refer to all components of the biopsychosocial perspective according to the ICF (Figure 3).

According to our analyses, the current state of health and sensation of pain (in particular pain at rest) at admission to inpatient rehabilitation are strong predictors regarding RTW 78 weeks after discharge. Patients who rated a state of health >53 (score: 0–100, higher values represent a better state of health) had an 82% higher odds (*p* = 0.0221; CI [1.10;3.07]), and patients who stated higher levels of pain at rest (score >26; score: 0–100, higher values represent more pain) had a 55% reduced odds (*p* = 0.0015; CI [0.27;0.73]) of having returned to work 78 weeks after discharge, respectively.

The professional sector, or more precisely, working in the construction, architecture, surveying, and building services engineering, is a strong predictor related to RTW. Among our study collective, being in this professional sector is associated with a 53% reduced odds (*p* = 0.0063; CI [0.28;0.81]) for RTW compared with patients not in this sector. Furthermore, individuals who stated financial concerns at admission to rehabilitation and persons who reported ongoing legal disputes related to the accident were less likely to be able to work 78 weeks after discharge (54% reduced odds, *p* = 0.0064; CI [0.26;0.81] and 57% reduced odds, *p* = 0.0004; CI [0.27;0.69]).

Of the five personality traits assessed by BFI-10, Conscientiousness and Neuroticism (range: 1–5, higher scores indicate a more pronounced personality trait) remained significant predictors in the model. An increase of 1 in the Conscientiousness score reduced the odds of RTW by 31% (*p* = 0.0315; CI [0.49;0.96]), whereas an increase of 1 in the Neuroticism score increased the odds of RTW by 36% (*p* = 0.0099; CI [1.08;1.73]).

The bivariate regression tree revealed 53 as a split value regarding life satisfaction preaccident (score: 0–100). Patients who rated their preaccident life satisfaction better at admission to inpatient rehabilitation, i.e., who scored >51, had a 67% reduced odds for RTW compared to patients who rated their preaccident life satisfaction as ≤51 (*p* = 0.0187; CI [0.12;0.78]).

We identified demand for pension claims as a highly significant predictor for RTW 78 weeks after discharge. Confirming the statement “I think I will probably apply for / get a pension in the near future” resulted in a 93% reduced odds of RTW (*p* = 0.0001; CI [0.03;0.30]) in the included study population.

Further results of the multiple logistic regression analysis are presented in Table 3.

**Table 3:** Prediction model of RTW at 78 weeks after discharge from initial inpatient rehabilitation (t6).

Predictors of RTW^a^	Relevant group	Compared group	Odds Ratio (OR)	95%-Confidence interval OR	*p*-value
General Health	“Good” vs. “Fair” / “Poor” vs.	“Excellent” / “Very good”	0.90 0.56	(0.48;1.64) (0.30;1.02)	0.7245 0.0633
Current state of health **(Score 0–100)**^b^	Score >53 vs.	Score ≤53	1.82	(1.10;3.07)	**0.0221**
Sensation of pain **(Score 0–100)**^b^	Pain at rest >26 vs. Pain under strain >35 vs.	Score ≤26 Score ≤35	0.45 0.59	(0.27;0.73) (0.31;1.11)	**0.0015** 0.1110
Limitations and restrictions in activities and participation (disability) **(Score 0–100)**^b^	Score >28 vs.	Score ≤28	0.56	(0.27;1.08)	0.0991
Professional sector	Construction, architecture, surveying and building services engineering vs.	Professional sector not selected	0.47	(0.28;0.81)	**0.0063**
Ongoing legal disputes	Legal disputes currently still ongoing vs.	No ongoing legal disputes	0.43	(0.27;0.69)	**0.0004**
Financial concerns (assets)	Financial concerns vs. No statement provided vs.	No financial concerns	0.46 0.56	(0.26;0.81) (0.30;1.06)	**0**.**0064** 0.0685
Personality traits **(Score 1–5)**^c^	Factor Neuroticism increased by 1 point		1.36	(1.08;1.73)	**0.0099**
	Factor Conscientiousness increased by 1 point		0.69	(0.49;0.96)	**0.0315**
Life satisfaction pre-accident **(Score 0–100)**^b^	Score >51 vs.	Score ≤51	0.33	(0.12;0.78)	**0.0187**
Attitude to life **(Work as an important purpose in life)**	“Rather applicable” vs. “Fully applicable” vs.	“Neither nor applicable” to “(rather) not applicable”	1.94 1.13	(0.94;3.90) (0.56;2.20)	0.0661 0.7320
Demand for pension claim	“I think I will apply for/get a pension in the near future.” vs.	Response option not selected	0.09	(0.03;0.30)	**0.0001**

OR intercept: 156.24.

Significant *p*-values (*p*-value <0.05) are in bold. OR in red indicates reduced odds of RTW 78 weeks after discharge from inpatient rehabilitation. OR in green indicates increased odds of RTW 78 weeks after discharge from inpatient rehabilitation.

^a^ Assessed at admission to initial inpatient rehabilitation.

^b^ A higher score indicates more pain, a higher level of disability (i.e., more limitations and restrictions in activities and participation), a higher life satisfaction preaccident, and a better current state of health.

^c^ A higher score indicates a more pronounced factor.

**Figure 4:**
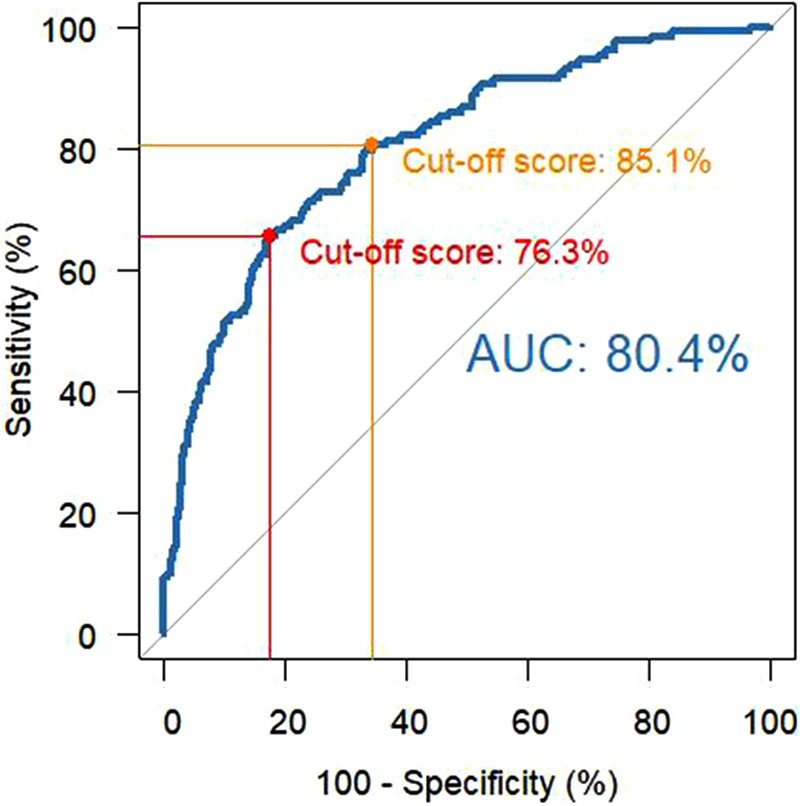
ROC curve (including AUC) and prediction accuracy on the training data for both cutoff scores [red: 76.3%, orange: 85.1%)].

A predicted probability for RTW based on the multiple logistic regression model of 76.3% was revealed as the optimal cut-off score based on the ROC curve (AUC = 80.4%), while an alternative cut-off score of 85.1% led to high sensitivity of at least 80% on the training data set (Figure 4).

For both cutoff scores, sensitivity, specificity, PPV, and NPV for the training data set and the validation data set are provided in Table 4.

**Table 4:** Sensitivity, specificity, PPV, and NPV for both cutoff scores for the training data set and the validation data set.

Cut-off score (%)	Data set	Sensitivity (%)	Specificity (%)	PPV (%)	NPV (%)
76.3	Training data set	65.6	82.5	46.4	91.2
	Validation data set	40.0	79.	31.6	84.7
85.1	Training data set	80.5	65.8	35.2	93.6
	Validation data set	53.3	63.5	25.8	85.1

## Discussion

This paper reports on the results of a multicenter longitudinal study conducted as part of the icfPROreha project. Our objective was to identify factors—taking into account a biopsychosocial perspective—relevant to the prediction of RTW of individuals with severe musculoskeletal injuries 78 weeks after discharge from inpatient trauma rehabilitation. Thus, we seek to contribute to a better understanding of the complexity of RTW after severe musculoskeletal injuries, detached from a purely biomedical approach.

Studies have shown that whether someone returns to work after an injury does not necessarily depend only on the type of injury but on multiple factors (5, 11–15, 27, 28). For example, poor mental health and pain severity, as well as strong social relationships and marked social functioning, were identified as predictors associated with RTW. Furthermore, personal factors such as occupation, age, and education also emerged as relevant aspects influencing RTW (4, 5, 11, 13, 15, 27–29). This highlights the need to consider environmental, personal, social, and psychological factors to address the complexity of RTW. A review of the scientific literature shows that research activities on RTW after traumatic injuries increasingly take into account a holistic approach, i.e., the consideration of the person with their professional, social and personal background (8, 11, 16, 30, 31). However, there are also considerable differences in the methodological and conceptual implementation of these studies, such as the definition of the outcome and duration of follow-up, limiting the comparability of the results (4, 5, 8). Nevertheless, the diversity and growing number of high-quality studies provide evidence and contribute to emphasizing the importance of a more holistic approach to patient-centered treatment and rehabilitation (32). This is critical because even though the biopsychosocial understanding of functioning and disability is now used in some areas of medical practice, such as rehabilitation or adolescent medicine (33), it still is almost unknown in other settings, such as acute care and surgery (34). The biomedical model, with its exclusive emphasis on the physical aspects of the disease, continues to be the predominant conceptual approach to medical work in this field (33). Yet, the idea of expanding the traditional biomedical approach has generated a positive response among those health professionals who prioritize patient care and treatment that is inherently comprehensive in its understanding of health and illness, and that is more consistent with the actual experiences of those who seek treatment (33).

In this study, we assessed a wide range of factors as potential predictors of RTW at admission to inpatient rehabilitation to cope with the biopsychosocial understanding of functioning and disability. Considering our results, it is advisable to distinguish between modifiable and generally nonmodifiable predictors because appropriate interventions should primarily target modifiable factors to avoid prolonged delay of RTW. Based on our data, 11 predictors of RTW were identified, which not only refer to functioning but also include environmental and personal factors. Predictors that reduce the odds of RTW are lower general health, higher level of pain and disability besides working in the construction sector, having stated ongoing legal disputes, and reporting financial concerns. Furthermore, being a very conscientious person, showing a higher overall life satisfaction before the accident and assuming to receive or apply for a pension in the near future reduced the odds for RTW in our population. Predictors that increase the odds of RTW are a higher score in the current state of health as well as considering work as an important purpose in life and having a higher score in the personality trait neuroticism.

Our results regarding predictors for RTW related to functioning, i.e., sensation of pain and level of disability, are in line with scientific research on RTW following a traumatic injury (4, 8, 10, 15, 35). Whereas a direct comparison of the results is limited, as time points of assessment (i.e., predictors and outcome measured) do not exactly match the time points used in our study. In their systematic review of biopsychosocial prognostic factors for RTW after acute orthopedic trauma, Duong et al. (8) reported strong to moderate evidence for a high level of pain and delayed RTW in the late stage (i.e., >6 months from injury), a result that is also reflected in a systematic review on barriers and facilitators associated with RTW following road traffic musculoskeletal injuries, published by Abedi et al. (4). However, none of the studies addressed a differentiated consideration with respect to pain at rest and pain under stress. Our analyses revealed the level of pain at rest—with a rather low threshold value of 26 points—to be the more crucial predictor (*p*-value: 0.0015). From a clinical point of view, the result is very plausible, and it provides valuable evidence that even low-level pain at rest should not be underestimated. In close contact with the patient, pain can be addressed at an early stage, for example, through appropriate pain communication, the involvement of a pain therapist, the use of passive therapies, or the administration of pain medication.

We further identified a number of contextual factors such as working in the construction sector (36), reporting ongoing legal disputes (14), experiencing financial concerns, and being a conscientious person as significantly reducing the chance for RTW 78 weeks after discharge from rehabilitation, some of which, however, must be considered as generally nonmodifiable predictors. A personality trait (e.g., conscientiousness) will be difficult to change, but it can be addressed with targeted interventions. In counseling sessions, for example, the patient is informed about the healing process that can realistically be expected, and it is conveyed that RTW is possible even without achieving the “perfect” preinjury status. Experience with work-related activities can be initiated at an early stage by involving occupational specialists. We found financial concerns at admission to be a relevant predictor for no RTW. It would be reasonable to assume that individuals with financial concerns would return to work earlier to avoid risking their jobs. We are not aware of further supporting literature regarding our findings; however, it has been shown that experiencing financial problems is a common burden after a traumatic injury (37–39). Murphey et al. reported an incidence of 88% for financial toxicity (i.e., lost wages, forced unemployment, financial burdens) within 1 year after injury. Considering our results from this point of view, an early assessment of the financial burden seems to be indispensable to be able to intervene at an early stage with the appropriate interventions and thus avoid long-lasting phases of no RTW.

To ensure optimized care, it is particularly important to identify at an early stage those individuals who are at risk for prolonged or no RTW. Thus, the rehabilitative treatment and measures can be optimized, for example, by applying the appropriate interventions or by involving specific health professions. Our analyses revealed a predicted probability for RTW at the admission of 76.3% as cutoff, i.e., in individuals with a predicted probability of RTW less than 76.3%, the predicted status will be “no RTW,” and conversely, in individuals with a predicted probability of RTW greater than 76.3%, the predicted status will be “RTW.” By assessing the identified predictors at admission to the first inpatient rehabilitation, 65.6% of the patients who will not RTW at 78 weeks after discharge from rehabilitation will be detected (sensitivity). Using the alternative cutoff of 85.1% would lead to an increase in the proportion of individuals being correctly identified as not going to RTW up to 80.5%. However, by doing so, the PPV would decrease from 46.4% to 35.2%, which means that of those receiving increased attention, only 35.2% would have an actual need. A PPV of 46.4% (or even 35.2%) may not seem particularly meaningful. However, it should be taken into account that the PPV cannot be close to 1 if the prevalence of the outcome is rather low (i.e., prevalence of no RTW in the current sample was 18.8%); accordingly, it is difficult to get a high PPV (10). As we already incorporated a validation data set to validate the results of the final logit model, we assume that a PPV of 35.2% can be considered a lower boundary and the exact PPV can be found between 46.4% and 35.2%.

The study is subject to certain limitations. Due to the extensive assessment, sufficient knowledge of the German language was required for participation in the study. Thus, a substantial proportion of patients who were not fluent in German had to be excluded. In particular, injured patients with a migration background and/or language comprehension problems, but also Germans with (functional) illiteracy, pose special problems for rehabilitation facilities and treatment providers. As predominantly clinics run by the employers' liability insurance association participated, mainly patients after occupational accidents were included. The payer is usually the German Social Accident Insurance (DGUV), which, in contrast to statutory health insurance, has a broader legal mandate and aims for rehabilitation “by all appropriate means.”

The particular strength of our study lies in the large number of patients included in the participating hospitals and the broad database available for the analyses. Due to the small number of missing values, both for the potential predictors and the outcome RTW, the data of almost all included patients could be considered in our analyses.

In general, there is still a lack of studies that comprehensively examine functioning and contextual factors related to RTW in individuals with traumatic injuries. Our study results support, however, the need for a holistic biopsychosocial view on functioning and contextual factors to further strengthen person-centered treatment and optimize guided rehabilitation (8, 11, 16, 30–32). Our results not only show that it is essential to consider contextual factors to predict RTW after severe musculoskeletal injuries but also provide insights into which contextual factors should be used to predict RTW at an early stage. With an outcome prognosis, based on the identified predictors, patients with a possible increased need for monitoring and optimization in the rehabilitation process can be identified already at the beginning of the rehabilitation intervention. This outcome prediction can be used in the clinics' rehabilitation departments, provided that applications for standardized assessment of functioning and contextual factors are available in the clinics.

Thus, knowing the relevant predictors allows recommendations for action to be developed to address these factors influencing RTW. Health care professionals to develop targeted interventions and strategies to prevent no RTW at an early stage. Recommendations for strategies can not only refer to the phase of inpatient rehabilitation but can also start at an early stage, e.g., after completion of acute care. Insurer specialists such as rehab managers can identify cases at risk of delayed RTW in good time and initiate the appropriate measures. In the interaction of all those involved in care, it is thus possible to prevent the accident victim, who wants to recover as quickly as possible, from becoming chronically ill.

## Data Availability

The raw data supporting the conclusions of this article will be made available by the authors without undue reservation.
