# Supplementary material for: ICF-based prediction of return to work after trauma rehabilitation: Results of the icfPROreha study in patients with severe musculoskeletal injuries
Source: Front Rehabil Sci. 2022 Sep 1;3:960473. doi: 10.3389/fresc.2022.960473 (PMC9474731; doi:10.3389/fresc.2022.960473)
Supplement: Supplementary file 1 [file Table_1_v1.docx]

Supplementary Table: Overview of variables, questions, response options and recoding.

| **Variables**  **(potential predictors)** | **Questions / Descriptions** | **Instruments / Source** | **Response options** | **Recoding** |
| --- | --- | --- | --- | --- |
| **Health Problem** |  |  |  |  |
| **General health** | In general, would you say your health is: | Short-Form 36 (SF-36) (1), Item 1 | 1 = excellent  2 = very good  3 = good  4 = fair  5 = poor | 1 = excellent / very good (1, 2)  2 = good (3)  3 = fair / poor (4, 5) |
|  | Body Mass Index: weight in kg / height in m^2 | -- | weight [kg]  height [m] | 1 = underweight / normal weight (BMI < 25)  2 = overweight (BMI: 25-30)  3 = obesity (BMI ≥ 30) |
| **Current state of health** | Your health today. | EuroQol five dimensions five levels (EQ-5D-5L) (2), visual analog scale (EQ-VAS) | score [0 = worst health you can imagine to 100 = best health you can imagine] | -- |
| **Pre-existing conditions (comorbidity)** | 14 Items (e.g. Cardiovascular disease) | Work Ability Index (WAI) short form (3), Item 3 | 2 = yes, own opinion  1 = yes, physician’s diagnosis  0 = no | 0 = no physician’s diagnosis  1 = one physician’s diagnosis  2 = two physician’s diagnoses  3 = three or more physician’s diagnoses |
| **Type of injury** |  |  |  |  |
| - Extensive or deep injuries of the skin and soft tissue mantle; amputation injuries; muscle compression syndromes (compartment syndromes); thermal or chemical damage - Injuries to the great vessels - Severe chest or abdominal injuries with organ involvement including kidneys or urinary tract - Complex fractures of the large tubular bones, especially multiple or open fractures - Severe injuries to large joints - Severe injuries to the hand - Complex fractures of the facial skull and torso skeleton - Multiple injuries with severe manifestations | VAV-Numbers | Index of Injuries (VAV) of DGUV and SVLFG (4) | 0 = injury not present  1 = injury present | -- |
| **Severity of injury** | VAV-Numbers | Index of Injuries (VAV) of DGUV and SVLFG (4) | 1 = one diagnosis: no severe injury  2 = one diagnosis: severe (S) injury  3 = two diagnoses: no severe injury  4 = two diagnoses: at least one severe (S) injury  5 = > 2 diagnoses: no severe injury  6 = > 2 diagnoses at least one severe (S) injury | -- |
| **Timely diagnosis** | Medical assessment | -- | [date of diagnosis] | -- |
| **Complications in healing process** | VAV-Number 11 | Index of Injuries (VAV) of DGUV and SVLFG (4) | 0 = complication not present  1 = complication present | -- |
| **Addictive behavior** |  |  |  |  |
| - Smoking Status | Do you smoke? | German Health Update (GEDA, RKI) (5), Question 112 | 1 = yes, daily  2 = yes, occasionally  3 = no, not any more  4 = never smoked | 0 = non-smoker (3, 4)  1 = smoker (1, 2) |
| - Heavy Drinking | 3 items (e.g. How often do you drink alcoholic beverages?) | Alcohol Use Disorders Identification Test (AUDIT-C) (6) | according to AUDIT-C | 0 = no risk  1 = increased risk for alcohol-related disorder  2 = risky alcohol consumption  3 = no information |
| - Gaming | 5 Items: (e.g. On average, how much time do you spend playing games on your computer, game console, smartphone/tablet or surfing the Internet for private purposes ("indeterminate surfing") on a normal working day? | icfPROreha project group | For example:  0 = less than 60 minutes  1 = 1 to less than 2 hours  2 = 2 to less than 3 hours  3 = 3 to less than 4 hours  4 = 4 to less than 5 hours  5 = 5 or more hours  6 = no information | Game-Score [0 to 4.6]; mean calculated (last response option considered as missing value) |
| **Functioning** |  |  |  |  |
| **Energy and drive functions** | Due to my physical impairment, I am less adventurous than before. | Patient Questionnaire for Assessment of Rehabilitation Motivation (PAREMO-20) (7), Item 1 | 1 = not true  2 = rather not true  3 = rather true  4 = true | -- |
| **Emotional functions** | 4 items (Over the last two weeks, how often have you been bothered by the following problems? E.g. feeling nervous, anxious or on edge) | Patient Health Questionnaire (PHQ-4) (8) | 0 = not at all  1 = several days  2 = more than half the days  3 = nearly every day | Sum score (0 to 12) was categorized:  0 = score < 3  1 = score ≥ 3 to < 6  2 = score ≥ 6 to < 9  3 = score ≥ 9 |
| **Sensation of pain** |  |  |  |  |
| - Pain at rest | How severe is your pain at rest? | Visual analog scale | [0 = no pain to 100 = excruciating pain] | -- |
| - Pain under stress | How severe is your pain under stress? | Visual analog scale | [0 = no pain to 100 = excruciating pain] | -- |
| **Functions of the cardiorespiratory system** | Did you exercise regularly before the accident? | National recommendations for physical activity and promotion of physical activity (BZgA) (9) | 0 = no, no regular exercise  1 = yes, moderate endurance-oriented exercise (e.g., fast walking) for less than 150 minutes per week  2 = yes, moderate endurance-oriented exercise (e.g., fast walking) for at least 150 minutes per week  3 = yes, intense endurance-oriented exercise (e.g., fast cycling or running) for less than 75 minutes per week  4 = yes, intense endurance-oriented exercise (e.g., fast cycling or running) for at least 75 minutes per week  5 = yes, muscle-strengthening physical activity (e.g., exercise) for fewer than 2 days per week  6 = yes, muscle-strengthening physical activities (e.g., exercises) for at least 2 days per week | 0 = no sport (0)  1 = sport, less than recommendation (1,3,5)  2 = sport according to recommendation (2,4,6) |
| **Structure of upper extremity**   - Upper extremity (including shoulder) | Medical assessment | According to: Abbreviated Injury Scale (AIS) – body regions (10) | 0 = body region not affected  1 = body region affected | -- |
| **Looking after one’s health** | How much do you care about your health in general? | German Health Update (GEDA, RKI) (5), Question 134 | 1 = very much  2 = much  3 = moderate  4 = less  5 = not at all | 1 = less / not at all (4, 5)  2 = moderate (3)  3 = very much / much (1, 2) |
| **Limitations and restrictions in activities and participation (disability)** | 12 Items (In the past 30 days, how much difficulty did you have in…: e.g. Taking care of your household responsibilities?) | WHO Disability Assessment Schedule 2.0 (WHODAS 2.0) (11) | 1 = non  2 = mild  3 = moderate  4 = severe  5 = extreme / cannot do | score [0 = no disability to 100 = complete disability] |
| **Environmental Factors** |  |  |  |  |
| **Type of accident - work or leisure accident (nc)** | Please answer some questions about your accident: I had a ... | icfPROreha project group | 1 = work accident  2 = commuting accident  3 = Leisure/sports accident  4 = Traffic accident  5 = Home/garden accident | 1 = work accident (1, 2)  2 = no work accident (3-5) |
| **Professional sector (e590)** |  |  |  |  |
| - Agriculture, forestry, animal husbandry and horticulture - Raw material extraction, production and manufacturing - Construction, architecture, surveying and building services engineering - Natural science, geography and information technology - Transport, logistics, protection and security - Commercial services, goods trade, distribution, hotel and tourism - Business organization, accounting, law and administration - Health, social services, teaching and education - Language, literature, humanities, social and economic sciences, media, arts, culture and design - Military | In which professional sector do you work? | German Classification of Occupations 2010 (12) | 0 = no  1 = yes | -- |
| **Ongoing legal disputes (e550)** | Are legal disputes still on-going in connection with the accident? | icfPROreha project group | 0 = no  1 = yes | -- |
| **Treatment: time from accident to admission to inpatient rehabilitation (e580)** | Calculation (difference) | icfPROreha project group | [date of accident; date of admission to inpatient rehabilitation] | -- |
| **Treatment: time from end of acute treatment to onset of post-acute treatment (e580)** | How many days were there between your discharge from the acute hospital and the start of rehabilitative measures (e.g. physiotherapy)? | icfPROreha project group | [days] | -- |
| **Treatment: Type of post-acute treatment (e580)**   - Physiotherapy - Occupational therapy - Massage / Lymphatic drainage - Medical training therapy - Psychological counseling / therapy - Other treatment - None | What treatments / interventions did you receive after discharge from the acute care hospital? | icfPROreha project group | 0 = no  1 = yes | -- |
| **Information about injury and prognosis by healthcare professionals (e355)** | Do you feel adequately informed about your injury and the consequences of injury? | icfPROreha project group | 0 = no  1 = yes | -- |
| **Availability of case management / coordination (e580)** | Do you already have personal contact with an occupational aide, rehab manager, or insurance case manager? | icfPROreha project group | 0 = no  1 = yes | -- |
|  | Has a goal agreement / rehab plan been completed? | icfPROreha project group | 0 = no  1 = yes | -- |
| **Financial concerns (assets) (e165)** | Do you currently have financial difficulties? | icfPROreha project group | 0 = no  1 = yes  2 = no information | -- |
| **Social insurance benefits (e570)** | Do you expect additional benefits from a private insurance? | icfPROreha project group | 0 = no  1 = yes  2 = no information | -- |
| **Support by family and friends (e310, e315, e325)** | How much do you feel supported by your family and friends? | icfPROreha project group | 1 = not at all  2 = somewhat  3 = moderately  4 = quite  5 = very | 1 = not at all to moderate (1, 2, 3)  2 = quite (4)  3 = very (5) |
| **Support from professional environment (employer, colleagues)** **(e325, e330, e335)** | How much do you feel supported by your company and/or colleagues? | icfPROreha project group | 1 = not at all  2 = somewhat  3 = moderately  4 = quite  5 = very | 1 = not at all to moderate (1, 2, 3)  2 = quite (4)  3 = very (5) |
| **Stressful life events (nc)** | Has there been at least one stressful life event (e.g. death of spouse/partner/family member, divorce or separation from spouse/partner, illness of a family member, dismissal without notice, unemployment, change in financial situation) in the last 12 months? | icfPROreha project group | 0 = no  1 = yes | -- |
| **Personal Factors** |  |  |  |  |
| **Age at admission** |  | --- | [in years] | -- |
| **Gender** |  | --- | 1 = female  2 = male | -- |
| **Family situation** |  | icfPROreha project group |  |  |
| - Living situation | Do you live… |  | 1 = alone  2 = with others | -- |
| - Social burden | How many financially dependent children do you have? |  | [number] | -- |
|  | Are there any special circumstances in your family that burden you? |  | 0 = no  1 = yes, care for a relative  2 = yes, single parent  3 = yes, other | -- |
| **Education** | What is your highest general school leaving certificate? | German General Social Survey (ALLBUS) 2016 (13), question F056 | 1 = pupil  2 = no graduation  3 = 8th or 9th grade  4 = 10th grade  5 = entrance qualification for a university of applied sciences  6 = higher education entrance qualification  7 = other graduation / free text | 1 = no graduation (2)  2 = graduation up to grade 9 (3)  3 = graduation up to grade 10 (4)  4 = graduation up to grade 12 (5, 6)  (free text (7) was allocated to other categories; pupil was omitted) |
| **Vocational training** | Do you have a completed vocational training? | icfPROreha project group | 0 = no  1 = yes | -- |
| **Cultural background** | What is/are your native language(s)? | icfPROreha project group | 1 = German  2 = Turkish  3 = Polish  4 = Russian  5 = Italian  6 = other | 0 = German  1 = other |
| **Social status** |  |  |  |  |
| - Income | What is the monthly net income of your household? [€] | According to: German General Social Survey (ALLBUS) 2016 (13), question F115 | 1 = < 900  2 = 900 to < 1300  3 = 1300 to < 1700  4 = 1700 to < 2300  5 = 2300 to <3200  6 = 3200 to < 4000  7 = 4000 to < 5000  8 = ≥ 5000  9 = no information | 1 = below 1700 (1-3)  2 = 1700 to < 2300 (4)  3 = 2300 to <3200 (5)  4 = 3200 or more (6-8)  5 = no information (9) |
| - Main earner | The household income corresponds to my personal income | icfPROreha project group | 0 = no  1 = yes  2 = no information | -- |
| **Employment status** |  |  |  |  |
| - Situation pre-accident | What was your professional situation before the accident? | icfPROreha project group | 1 = fully employed  2 = partly employed (half-day, hourly)  3 = unable to work  4 = registered jobseeker  5 = other | 1 = not working (3, 4);  2 = partly employed (2);  3 = fully employed (1);  "other" was assigned, if possible; |
| - Employment type | In your current job, are you...? | icfPROreha project group | 1 = …self-employed (own business, own office or similar)  2 = … dependent employed (working as worker, employee or civil servant) | -- |
| **Subjective prognosis on RTW** | Do you think that, based on your current state of health, you will still be able to do your current job in a year's time? | icfPROreha project group | 1 = unlikely  2 = not sure  3 = very likely | -- |
| **Demand for pension claim** | I think I will probably apply for / get a pension in the near future. | Screening Instrument for the Access to Work-Related Multimodal Rehabilitation (SIMBO) (14), Item SI 6, response option 5 | 0 = no  1 = yes | -- |
| **Inability to work before the accident** | How many weeks in the last 12 months before the accident were you unable to work in total? | icfPROreha project group | [weeks] | -- |
| **Personality traits**  - Extraversion  - Neuroticism  - Openness  - Conscientiousness  - Agreeableness | 10 Items (e.g. I see myself as someone who is reserved), 2 for each personality factor | Big Five Inventory (BFI) (15) | 1 = disagree strongly to 5 = agree strongly | 5 BFI-10 Scales: Extraversion, Neuroticism, Openness, Conscientiousness, Agreeableness [1 to 5; higher scores indicate a more pronounced personality factor] |
| **Self-efficacy** | 3 items (e.g. I can rely on my abilities in difficult situations) | Short Scale for Measuring General Self-efficacy Beliefs (ASKU) (16) | 1 = disagree strongly to 5 = agree strongly | score [1 to 5; higher scores indicate a more pronounced factor] |
| **Attitude to life** (work as an important purpose in life) | Work is an important part of my life. | icfPROreha project group | 1 = does not apply at all to 5 = fully applies | 1 = neither nor to not applicable (1,2,3)  2 = rather applicable (4)  3 = fully applicable (5) |
| **Appraisal of the consequences of the accident** | How do you rate the consequences of the accident? | icfPROreha project group | 1 = not at all burdensome  2 = little burdensome  3 = somewhat burdensome  4 = quite burdensome  5 = extremely burdensome | 1 = somewhat to not at all burdensome (1,2,3)  2 = quite burdensome (4)  3 = extremely burdensome (5) |
| **Life satisfaction pre-accident** | Before the accident, how satisfied were you - all in all - with your life? | According to: German General Social Survey (ALLBUS) 2016 (13), question F129 | visual analog scale [0 = completely dissatisfied to 100 = completely satisfied] | -- |
| **Resilience** | 13 items (e.g. I like myself.) | Resilience Scale (RS-13) (17) | 1 = disagree to 7 = agree | sum score (13-91)  13-66 = low  67-72 = moderate  73-91 = high |
| **Coping / dealing with the injury** | How well can you cope with the aftermath of your injury? | icfPROreha project group | 1 = very good  2 = good  3 = moderate  4 = poor  5 = very poor | 1 = very good / good (1,2)  2 = moderate (3)  3 = poor / very poor (4,5) |
| **Disease gain** | 4 items (e.g. When I'm feeling bad, someone is more likely to take care of me than usual.) | Patient Questionnaire for Assessment of Rehabilitation Motivation (PAREMO-20) (7), Items 2, 7, 14, 19 | 1 = not true  2 = rather not true  3 = rather true  4 = true | -- |

**eReferenzen**

1. Ware J, Sherbourne CD. The Mos 36-Item Short-Form Health Survey (Sf-36). I. Conceptual Framework and Item Selection. *Med Care* (1992) 30(6):473-83.

2. The-EuroQol-Group. Euroqol - a New Facility for the Measurement of Health-Related Quality of Life. *Health Policy* (1990) 16(3):199-208. doi: <https://doi.org/10.1016/0168-8510(90)90421-9>.

3. Ilmarinen J. The Work Ability Index (Wai). *Occup Med* (2007) 57(2):160. doi: 10.1093/occmed/kqm008.

4. Deutsche Gesetzliche Unfallversicherung Und Sozialversicherung Für Landwirtschaft Forsten Und Gartenbau, Verletzungsartenverzeichnis: Verletzungsartenverzeichnis Mit Erläuterungen Unter Einschluss Des Schwerstverletzungsartenverfahrens (Überarbeitete Version 2.0, Stand 1. Juli 2018) (2018) [cited 2022 17.05.]. Available from: <https://www.dguv.de/medien/landesverbaende/de/med_reha/documents/verletz3.pdf>.

5. Robert-Koch-Institut. Fragebogen Zur Studie „Gesundheit in Deutschland Aktuell“ Geda 2014/2015-Ehis. *J Health Monit* (2017) 2(1):105-35. doi: 10.17886/RKI-GBE-2017-014.

6. Bush K, Kivlahan DR, McDonell MB, Fihn SD, Bradley KA, Project ftACQI. The Audit Alcohol Consumption Questions (Audit-C): An Effective Brief Screening Test for Problem Drinking. *Archives of Internal Medicine* (1998) 158(16):1789-95. doi: 10.1001/archinte.158.16.1789 %J Archives of Internal Medicine.

7. Hafen K, Jastrebow J, Nübling R, Bengel J. Entwicklung Eines Patientenfragebogens Zur Erfassung Der Reha-Motivation (Paremo). *Rehabilitation* (2001) 40:3-11.

8. Kroenke K, Spitzer RL, Williams JB, Löwe B. An Ultra-Brief Screening Scale for Anxiety and Depression: The Phq-4. *Psychosomatics* (2009) 50(6):613-21. Epub 2009/12/10. doi: 10.1176/appi.psy.50.6.613.

9. *Nationale Empfehlungen Für Bewegung Und Bewegungsförderung*. FAU Erlangen-Nürnberg: Alfred Rütten & Klaus Pfeifer (2016).

10. States JD, Huelke DF. *The Abbreviated Injury Scale 1980 Revision*. American Association for Automotive Medicine (AAAM). Morton Grove, IL, Proceedings (1980).

11. Ustun TB, Kostanjesek N, Chatterji S, Rehm J, World Health O. *Measuring Health and Disability : Manual for Who Disability Assessment Schedule (Whodas 2.0) / Edited by T.B. ÜStüN, N. Kostanjsek, S. Chatterji, J.Rehm*. Geneva: World Health Organization (2010).

12. Klassifikation Der Berufe (Kldb) 2010: Bundesagentur für Arbeit (BfA) - Statistik (2022) [cited 2022 25.05.]. Available from: <https://statistik.arbeitsagentur.de/DE/Navigation/Grundlagen/Klassifikationen/Klassifikation-der-Berufe/KldB2010/KldB2010-Nav.html>.

13. Allbus 2016: Fragebogendokumentation. GESIS – Leibniz-Institut für Sozialwissenschaften, (2016) Contract No.: 17.05.2022.

14. Streibelt M, Gerwinn H, Hansmeier T, Thren K, Müller-Fahrnow W. Simbo: Ein Screening-Instrument Zur Feststellung Des Bedarfs an Medizinisch-Beruflich Orientierten Maßnahmen in Der Medizinischen Rehabilitation - Analysen Zur Konstruktvalidität Und Prognosegüte. *Rehabilitation* (2007) 46:266-75. doi: 10.1055/s-2007-970583.

15. Rammstedt B, John OP. Measuring Personality in One Minute or Less: A 10-Item Short Version of the Big Five Inventory in English and German. *Journal of Research in Personality* (2007) 41(1):203-12. doi: <https://doi.org/10.1016/j.jrp.2006.02.001>.

16. Beierlein C, Kemper C, Kovaleva A, Rammstedt B. Short Scale for Measuring General Self-Efficacy Beliefs (Asku). (2013). doi: 10.12758/mda.2013.014.

17. Leppert K, Koch B, Brähler E, Strauss B. Die Resilienzskala (Rs) – Überprüfung Der Langfrom Rs-25 Und Einer Kurzform Rs-13. *Klinische Diagnostik und Evaluation* (2008) 1:226-43.
